# A Critical Appraisal of Adult Trigger Finger: Pathophysiology, Treatment, and Future Outlook

**DOI:** 10.1097/GOX.0000000000002360

**Published:** 2019-08-08

**Authors:** Nikolas Brozovich, Devandra Agrawal, Gangadasu Reddy

**Affiliations:** From Creighton University School of Medicine, Omaha, Nebr.

## Abstract

Trigger finger (TF) is a common referral to a hand surgeon, with people with diabetess being the most at-risk population. Abnormal thickening, scarring, and inflammation occur at the A1 pulley and flexor tendon, and histological changes correlate well with the clinical severity of TF. Corticosteroid injections decrease the thickness of the A1 pulley and are considered a first-line treatment. However, corticosteroids are only moderately effective, especially for people with diabetes. Patients may elect for surgery if nonoperative treatments prove ineffective; some may choose immediate surgical release instead. To release the A1 pulley, patients have the option of an open or percutaneous approach. The open approach has a greater risk of infection and scar tissue formation in the short run but an overall superior long-term outcome compared with the percutaneous approach.

**Methods:**

We critically reviewed the efficacy and cost-effectiveness of the treatment methods for TF through a comprehensive search of the PubMed Database from 2003 to 2019.

**Results:**

To reduce costs, while still delivering the best possible care, it is critical to consider the likelihood of success for each treatment method in each subpopulation. Furthermore, some patients may need to return to work promptly, which ultimately may influence their desired treatment method.

**Conclusions:**

Currently, there is no universal treatment algorithm for TF. From a purely financial standpoint, women without diabetes presenting with a single triggering thumb should attempt 2 corticosteroid trials before percutaneous release. It is the most cost-effective for all other subpopulations to elect for immediate percutaneous release.

With a lifetime risk of 2.6%, trigger finger (TF) is the fourth most common reason for referral to a hand surgery clinic.^25^ The average age of onset is 58, with women 2–6 times more at risk than men.^25^ Most commonly, the thumb and ring finger of the right hand are affected.^13^ Overuse, trauma, diabetes, and carpal tunnel syndrome are all risk factors for the development of TF. TF develops because of scarring and inflammation of the A1 pulley, the first of a 5-pulley system in hand (Fig. 1). Thickening of the A1 pulley and, to a lesser extent, the flexor tendon has been observed.^37^ Both stenosis of the A1 canal and nodules on the tendons at the bifurcation area of digitorum flexor superficialis can produce pain, cracking, and locking.^8^ The identifiable locking feature of TF is observed when an affected digit moves from flexion to extension. Pathological grading of the abnormal A1 pulley strongly correlates with the clinical severity of TF. This includes the presence of irregular connective tissue, chondroid metaplasia, and rounded nuclei of the inner layer. Hyaluronic acid, chondroitin sulfate, and proteoglycan accumulation are also associated with the severity of this syndrome.^60^

**Fig. 1.**
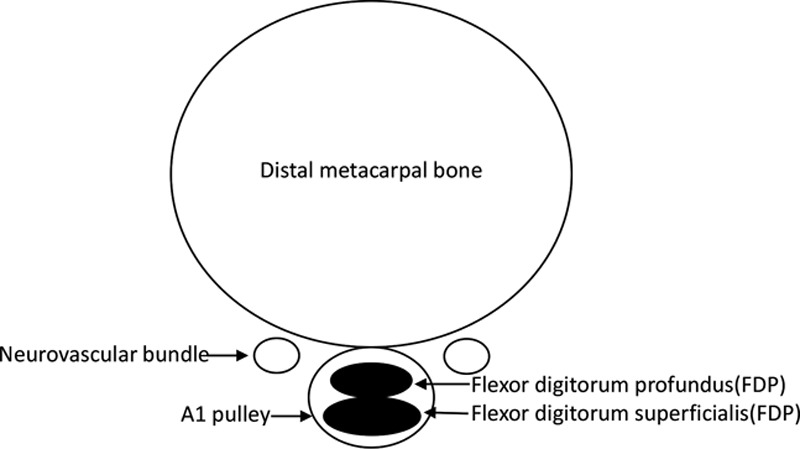
Cross-section of distal metacarpal bone.

The Quinnell grading system is used to assess clinical severity of TF (Table 1). Most studies regarding TF use symptom resolution to evaluate outcome, as opposed to using hand function tests. The Purdue Pegboard Test, Functional Dexterity Test, and Jamar Hydraulic Hand Dynamometer scores are valid tools for hand dexterity and strength. All moderately correlate with the Disabilities of Shoulder and Hand (DASH) questionnaire, but the Purdue Pegboard Test is the most sensitive to the clinical grading of TF.^25^

**Table 1.** Quinnell Grading

Grade	Clinical presentation
I	Uneven movement
II	Actively correctable
III	Passively correctable
IV	Fixed deformity

Common first-line treatments for TF include corticosteroid injections and occupational therapy. Both have shown to improve grip strength, pain, and frequency of triggering events. Patients are more satisfied with corticosteroid treatment, but corticosteroid injections show a greater rate of symptom recurrence after sixth month.^36^ If symptoms do not improve with conservative therapy, surgery is recommended to release the first annular pulley. There is no established algorithm for TF treatment, and management variation most likely can be attributed to the hand surgeon’s training.^42^

## INCIDENCE AND ASSOCIATED DISEASES

Overuse, diabetes, gout, acromegaly, renal disease, glycogen storage diseases, carpal tunnel syndrome, rheumatoid arthritis, and other rheumatoid and musculoskeletal disorders have been associated with TF,.^27,35^ Thyroid dysfunction, particularly hypothyroidism and thyrotoxicosis, have also been associated with TF.^5^ Lifetime risk of TF for the general population is 2.6% compared with 10% for people with diabetes.^15^ Others report rates of 1%–2% for the general population, and 10%–20% for people with diabetess; 25% of patients presenting with TF are diabetic.^62^ Furthermore, around half of patients with diabetes with TF will present with multiple digit involvement.^41^ The longer a patient has diabetes, and specifically a high HbA1c, the more likely they will be affected by a hand or shoulder disorder.^19,39^ An HbA1c level greater than 7% is an independent risk factor for the development of TF, but an HbA1c level beyond 7% does not further increase risk.^15^ One report showed 60% of TF cases in patients with diabetes to recover spontaneously, compared with only 20%–29% of all TF cases.^41^

Carpal tunnel release (CTR) has been associated with the development of TF; the most common finger that triggers following a CTR is the thumb.^1^ CTR is accomplished by the release of the flexor retinaculum; however, this leads to the bowstring effect. The bowstring effect theoretically causes an increased friction force on the flexor tendons, putting patients at risk for TF. Within the first 6 months, there is a 9.65-fold increase in risk, and overall a 3.63-fold higher risk, when compared with the general population.^27^ In addition, patients with diabetes are more likely to develop TF following a CTR compared with patients without diabetes. For patients with diabetes, 8% will develop TF within 6 months of a CTR, and 10% within a year, compared with 3% and 4%, respectively, for non-diabetics.^15^ CTR involving removal of the forearm fascia has a greater risk for postoperative TF compared with transverse carpal ligament release alone due to an increased entrance angle of the flexor tendons into the A1 pulley with resulting friction and ultimately triggering events.^1^ Research that supports an increased risk of TF following CTR uses the contralateral hand as a control. Carpal tunnel syndrome and TF have common pathologies and are often diagnosed concomitantly in the same hand. CTR may not increase the risk of developing TF in the operative hand, but rather there is an intrinsic risk of developing TF in hands with carpal tunnel syndrome.^61^

## TISSUE ARCHITECTURE

Ultrasonography has demonstrated that untreated TF has on average an A1 pulley thickness of 1.1 mm; 1 month following corticosteroid treatment, the A1 pulley decreased to 0.7 mm for intrasheath injection and 0.8 mm for extrasheath injection.^51^ Injection site does not significantly affect the outcome. In addition to a thicker A1 pulley, a thicker proximal region of the A2 pulley and flexor tendon is also characteristic of TF,^23^ and within a month of corticosteroid treatment, tendon thickness significantly decreases. Tendon thickness decreased from 4.1 mm (for intrasheath) and 4.0 mm (for extrasheath) to 3.9 and 3.8 mm, respectively; following corticosteroid treatment, both flexor digitorum tendon and A1 thickness were comparable to control values.^37^ It is speculated that corticosteroids have a more immediate effect on the A1 pulley than the tendon due to differences in tissue density. Tendon rupture may occur with repeated high-dose therapy.^53^

## HISTOPATHOLOGICAL CHANGES

The A1 pulley comprised 3 layers. The outer layer is a highly vascularized convex layer that is continuous with the tendon sheath. The inner 2 layers are avascular and function as the concave gliding surface for the tendon; the first layer contains cartilage-like cells, and the second has spindle-shaped fibroblasts with elongated nuclei and compact parallel regular collagenous bundles.^12,29^ The inner fibrocartilage becomes thinned and replaced by fibrous tissue in moderate TF.^12^ Pathological transformation begins with a myxoid matrix between collagen and evolves to an irregular distribution of chondromyxoid matrix with vascular hyperplasia. Normal elongated nuclei of fibroblasts are replaced by rounded nuclei of chondrocytes. The accumulation of hyaluronic acid, chondroitin sulfate, and proteoglycan is associated with syndrome severity. Pathologically, the highest grade of TF contains invasive chondroid metaplasia.^60^ Computer grading using abnormal tissue and round nuclei as the pathological parameters strongly correlates with clinical severity and pathological grading.^29^

Evidence also suggests tendinosis of the trigger tendons; tissue samples of the tendons in TF had a Movin Score 14.2 compared with 2.5 for normal finger tendons.^31^ Trigger tendons also show significant upregulation of collagen types 1a1 and 3a1, aggrecan, and bigylcan, and downregulation of MMP-3 and TIMP-3.^30^

The histopathology of the tenosynovium surrounding the A1 pulley is also abnormal. It was found that 61% of samples comprised hyaluronic acid producing chondrocytoid cells that express CD44 (a marker for synovial B cells), but not S100 (normal chondrocyte cell surface antigen). Additionally, a hypocellular collagen matrix is observed in 84% of TF tenosynovium. This edematous tissue most likely contributes to the pressure between the A1 pulley and the tendon, contributing to the progression of TF. Inflammatory infiltrate, increased vascularity, hyperplasia of synovial lining, and fibrin exudation, which are markers of synovitis, are present in only 5%, 37%, 37%, 21%, and 5% of tissue.^56^

## CURRENT TREATMENT

### Corticosteroids

Acute TF is treated with immobilization, ice application, and anti-inflammatory medications.^52^ If symptoms persist, injection with Triamcinolone, a synthetic corticosteroid, is the treatment of choice. Triamcinolone relieves symptoms in 83% of patients compared with 30% for dexamethasone.^38^ An appropriate dose of triamcinolone is 5–10 mg. No significant differences between the palmar proximal, palmar distal, and web space approaches have been observed.^46^ If symptoms do not resolve within 6 weeks of the initial injection, another corticosteroid injection can be administered.

Despite no histological evidence of inflammation at the A1 pulley, corticosteroids seem to reduce swelling of the A1 pulley.^38^ Studies have demonstrated that corticosteroid treatment reduces the synthesis of collagen type I and proteoglycans. Furthermore, there is a decrease in tenocyte proliferation, differentiation, viability, and metabolism. Under some conditions, corticosteroids can increase the synthesis of MMP-1 and MMP-13, which ultimately leads to further cleavage of collagen type 1.^53^

Success rate is reported to be 57% after a single injection, and 86% after a second injection within a 6-month follow-up period^49^. A retrospective review, in which average follow-up was 5.5 years, reported corticosteroid injections to relieve all symptoms in 79.7% of patients. Of those whose symptoms recurred, it was on average after 315 days.^50^ Others found that 69% of patients had complete remission of their symptoms with a median follow-up of 8 years; trigger thumbs had a success rate of 81%, and all other digits had a success rate of merely 56%.^7^ If patients live symptom free for 2 years following corticosteroid treatment, they likely will remain symptom free.^59^ Women who present with a single TF are most likely to have long-term success with a single corticosteroid injection; a study determined the 10-year success rate of women to be 56%, compared with 35% for men. For women and men with multiple TFs, long-term success after a single injection is 39% and 37%, respectively.^59^ Success rate for patients with diabetes has been reported as 57% compared with 72% for patients without diabetes.^7^ Corticosteroid injections are also believed to be less effective for patients with symptoms that have been present for over 6 months.^34^

### Alternative Nonsurgical Therapies

Other non-surgical treatments have shown to be less effective than corticosteroid injections.^2^. Custom-made night orthotic splints help reduce pain and disability in patients with low-grade TF with symptoms presenting for less than 6 months; recommendations are to immobilize the joint from 6 to 10 weeks.^32^ Joint splints are reported to have positive outcomes ranging from 50% to 93%.^57^ Similarly, others reported symptom resolution and an increase in grip strength in 66%–92% of patients who wore an MCP blocking orthosis for 3–9 weeks.^24^ Orthosis for MCP joints are more comfortable and have better outcomes than DIP splints. Just like splinting and corticosteroids, acupuncture has been shown to be more effective when TF first presents; it is believed to reduce inflammation of the synovial membrane of the sheath.^20^

Physiotherapy has also been shown to be a semieffective treatment for TF. Three months following the start of treatment, 68.6% of patients found their symptoms to improve compared with 97.4% of patients receiving steroid injections. The physiotherapy group had no symptom recurrence at a 6-month follow-up, while symptom recurrence was observed in the steroid injection group.^48^ Physiotherapy can serve as concurrent rehabilitation treatment method to improve symptom relief. Despite viable nonsurgical therapies, most patients will eventually require surgical release (Fig. 1; Table 1).^10^

### Surgery

After 2 failed steroid treatments, surgery is recommended.^52^ However, many surgeons and patients may forgo corticosteroid treatment and instead elect immediate percutaneous or open surgery. An open approach is the “gold standard,” in which a longitudinal incision is preferred to ensure complete release; in addition, it helps surgeons to identify a rare case of atraumatic rupture of the flexor digitorum profundus tendon.^3^ Open surgery has a success rate of 99% ^18^; the success rates for percutaneous release ranges from 74% to 94%.^16^ If the percutaneous release is unsuccessful, a second percutaneous release or an open procedure can be considered.^58^ Some studies report success rates of 98%–100% for percutaneous release. However, these studies used symptom resolution as a marker for success, but this does not accurately reflect patient satisfaction regarding grip strength and residual pain.^24^ One study found persistent pain in as many as 50% of patients who underwent percutaneous release.^3^

The percutaneous technique, compared with the open procedure, offers the advantage of being less invasive, which decreases the risk for infection, scar tissue formation, and finger stiffness.^47^ However, open release has a lower risk of iatrogenic nerve damage compared with percutaneous release.^18^ Regarding pain and patient satisfaction, percutaneous release groups seem to outperform open release surgery in the short run.^17^ One study found that patients undergoing ultrasound guided percutaneous release return to normal activities after 4.1 days compared with 17.8 days for an open approach^40^. Some studies find open release surgery to have superior long-term outcomes, while other studies see no difference in long-term outcomes between percutaneous and open release surgery.^17,26^

Many surgeons concurrently administer local steroid injections while performing a percutaneous release, but no significant difference in outcomes has been observed.^28^ During an open release, it has been reported that patients who receive presurgical local steroids have less postoperative swelling, and those on systemic steroids have faster postoperative symptom resolution.^6^

Typically, local anesthetics are used perioperatively, and NSAIDs and opioids are administered postoperatively. Around 30%–40% of patients suffer from moderate to severe pain 24–48 hours following the procedure. Marcaine alone has been shown to be more effective in controlling pain than lidocaine; Marcaine used in conjunction with Exparel, an extended release local anesthetic, is the most effective pain management strategy with only 50% of patients requiring additional pain medications.^22^

### Cost Analysis of the Pharmacological and Surgical Treatments

US health care costs make up 17% of the US GDP, and it continues to outpace inflation.^33^ Therefore, utilization of cost-effective practices without sacrificing patient care is imperative.^33^ Open release of the A1 ligament remains the most effective long-term treatment for TF. Splinting, physical therapy, and triamcinolone injections, in addition to percutaneous release, serve as more cost-effective first-line treatment methods. Usually, 2 steroid injections before surgery is the most cost-effective treatment strategy.^21^ Percutaneous release is more cost-effective compared with open release because it can be performed in the office.^43^ Only an anesthetic and disposable 18-gauge needle are required for this procedure, while open release requires sterilized equipment, a skin incision, suture, and surgical room.^35^ Percutaneous release in the clinical setting, with subsequent open release in an ambulatory surgical center for those who fail initial percutaneous release, has an attributed cost of $603. Open release in a hospital setting costs approximately $1,192, while open release in an ambulatory surgical center is $642.^14^ The cost of open release in an ambulatory surgical center utilizing the wide awake local anesthesia no tourniquet technique allows for a reduction in cost.^44^ The alternative would be monitored anesthesia care case in a hospital setting, which has the costs of anesthesia, additional materials, more time in the OR, and more time in the recovery room.^9^ Percutaneous release remains more cost-effective than open release in an ambulatory surgical center contingent upon a percutaneous success rate greater than approximately 91%. In 2013, only 5% of TF releases were percutaneous releases performed in a clinical setting, while 61% and 34% were open releases performed in an ambulatory surgical center and hospital, respectively.^14^

Corticosteroids have been shown to be less effective in people with diabetes, men, nonthumb digits, and multidigit cases.^41^ For patients with diabetes, an immediate percutaneous release in the clinical setting is the most cost-effective under the assumption that injection failure rate is at least 34%.^33^ Under this same assumption, patients with metabolic syndrome, which reports a corticosteroid injection failure rate of 49%, should also elect for percutaneous release as their initial treatment method from a cost-effective standpoint.^45^ Men with single digit involvement, men with multiple digit involvement, and women with multiple digit involvement should forgo corticosteroid injections from a purely financial standpoint with success rates of 35%, 37%, and 56%, respectively, as reported in a 10-year follow-up study.^59^ From a purely cost-analysis perspective, the only patients who should attempt 2 corticosteroid treatments before percutaneous release are women without diabetes who present with a single triggering thumb. Despite this, corticosteroids remain a viable first-line option, especially for those who do not wish to undergo surgery. On the other hand, some may prefer surgery as a first-line treatment due to a more predictable outcome and recovery time.^40^

## CONCLUSIONS AND FUTURE DIRECTIONS

In TF, abnormal swelling and inflammation have been noted in the flexor tendon and the A1 pulley. However, there does not seem to be an abundance of inflammatory infiltrate, but rather metaplasia. This abnormal response has contributed to a greater pressure between tendon and ligament, which like other musculoskeletal disorders is exacerbated by hyperglycemia. Further research into the etiology, pathology, and histological changes of TF will help to develop novel treatments. In addition, the establishment of an animal model would help evaluate tissue composition, treatment methods, and the role of diabetes in various musculoskeletal disorders.
